# Bibliography

**Published:** 1894-12

**Authors:** 


### Bibliography.

The Anatomy and Pathology of the Teeth. By C. F. W. Bödecker, D.D.S., M.D.S. With three hundred and twenty-five Illustrations. The S. S. White Dental Manufacturing Company, Philadelphia, 1894.

The readers of dental literature have heard from time to time that Dr. Bödecker had in preparation a work on dental pathology, and its appearance has been awaited, by some at least, with eager interest.

The author's well-earned reputation and long service in this direction led to the hope that at last we were to have a pathological work not only of scientific value but of practical importance.

The author has shown in his "decade of work" a patient and persistent effort to make a volume worthy the perusal and study of the intelligent dental practitioner; and in this he has certainly succeeded, for it is imagined very few of that class but will give its ideas close attention, though it is not to be expected that many workers in dental tissues will accept his conclusions.

The book opens with a description of the superior maxillary bones, followed by the inferior, characteristics of the teeth, articulation, and general anatomy. These cover forty-six pages.

With Chapter VIII. commences General Histology, and from this on through three hundred and one pages the author gives in detail his own views in regard to the development of dental tissues, together with those of Heitzmann, Abbott, Hart, etc. Much of this has been made familiar to dental students in articles prepared for dental periodicals. The long contest waged over the theories of Heitzmann, Bödecker, and Abbott may possibly be fought once again now that they are presented in a concrete form. In fact, the work seems to be in the nature of a challenge to the world at large in this direction. Space does not permit, even were it justifiable, a criticism of these theories. While there is much in them in accord with the writer's views, there is also much that he cannot accept. The charge frequently made, that the new views of the histology of tooth structure are based solely on drawings in which the personal equation is a prominent factor, will doubtless be repeated now that we have them in consecutive order, for the drawings are all by Heitzmann. This, as the writer views it, is a grave error. Drawing, however beautifully done, and Heitzmann is a master in this direction, cannot carry conviction. A few photomicrographs would have accomplished far more. After all that has been said antagonistically to the claims of a reticulum in tooth structure, it seems strange that the author should have allowed this book to go to press with no real evidence to prove the correctness of the theories advanced. It leads to the suspicion, whether justly founded or not, that either the slides cannot be represented or that they have no real existence. Those who know the author are not prepared to believe this; hence it would seem that no expense should have been spared to meet this demand.

Chapter XXI. begins what may be called the second part of the book, and opens with "Inflammation." With the exception of a few paragraphs this entire chapter is taken from a paper by William Atkinson, M.D., of New York, on the "Origin of Pus." Why the author should have given the views therein contained as being "based on the investigations" of Dr. Atkinson remains a mystery, as the reader will fail to find a record of personal investigation, but a good deal of history, and a large proportion decidedly out of harmony with modern ideas.

It is strange that the blunder of Atkinson should have been repeated here, wherein he stated in the original paper "that the emigration of the leucocytes had been seen in England by Walker and Wallace. If any such men existed in connection with this subject, begun by Döllenger in 1819, the writer is not aware of it. Williams, Addison, and Waller, from 1842 to 1846, were the distinctive English names connected with this work, and Waller so clearly proved the diapedesis of the leucocyte that, notwithstanding all the difficulties he had to contend against, his work stood ; so that when Conheim made his brilliant discovery in 1867, Waller's name was eventually joined with the latter as having equal merit in the work. The fact that the author accepts Atkinson's work makes him responsible for errors.

The Waller-Conheim theory of emigration of the leucocytes of the blood finds scant favor at the author's hands, as he evidently regards that of Heitzmann as covering all of truth on this question.

In the consideration of the pulp the author adopts, in part, Arkövy's classification (Diagnostik der Zahnkrankheiten), though he expressly disagrees with him on some points. This chapter is interesting and instructive, and altogether worthy of the author, although some exceptions might be taken to his divisions, which seem unnecessary and tending to complications.

In the chapter on Pericementitis this tendency to minute pathological subdivisions is manifest to a very objectionable degree, as he divides the non-purulent under six heads,—1, Acute marginal pericementitis; 2, acute circumscribed pericementitis; 3, acute apical pericementitis; 4, acute diffuse pericementitis; 5, chronic hyperplastic partial pericementitis; 6, chronic hyperplastic diffuse pericementitis,—"Purulent" under six heads, and "Secondary Forms" under four, making sixteen divisions in all.

It is not possible to see any good or sufficient reason, beyond the multiplication of words, for this arrangement. The division between purulent and non-purulent is very questionable; for, in the reviewer's opinion, there can be no inflammation without emigration of leucocytes, and this means a purulent condition more or less extended as the inflammation progresses. The author seems to recognize this, for he says, "Doubtless the largest accumulation of the inflammatory elements takes place in the immediate neighborhood of the blood-vessels, for in the midst of many nests capillaries can be traced. . . . This fact has been taken up as an argument by those who assert that the inflammatory nests are altogether due to emigration."

Among the number of subdivisions is included pyorrhœa alveolaris, under the name of "Chronic Purulent Marginal Pericementitis."

The author antagonizes recent theories of the gouty diathesis, for he says, "It cannot be denied that a superabundance of uric acid in the system may assist in producing pyorrhœa alveolaris, but it will not exert more influence in this direction than consumption, anæmia, nephritis, hepatitis, diabetes, arthritis, syphilis, typhoid, chronic diseases of the nervous system, etc."

In the chapter on Caries the author concedes the truth of Miller's views, but qualifies it by stating that "I am convinced that only by a combination of both the inflammatory and the bacteriological doctrines the full truth will be reached."

In the chapter on Erosion the author slurs over as of no moment all theories excepting those which have had gout as a basis, for he writes, "Much was written on this subject *without advancing anything new* till E. T. Darby, . . . in 1892, stated erosion is mostly found in teeth of patients who suffer more or less from gout." He acknowledges that E. C. Kirk read a paper before the First District Dental Society in 1886 in which he demonstrated that its origin was from acid mucoid secretions of the mouth. He, however, gives priority to J. Truman.

This is certainly unjust to the writers named. The ideas they advanced were based on actual investigations, and were new at the time they were advanced, and were the first to give an intelligent reason for this pathological condition. The attention of the author is called to the Proceedings of the Odontological Society of Pennsylvania for January, 1884, and to note on page 242, April number, of the INTERNATIONAL DENTAL JOURNAL for 1893, for Dr. Truman's views. It is not necessary here to repeat them; suffice it to say there is a marked difference between these and Dr. Kirk's, though both are in accord. He will find that subsequently Dr. Brubaker confirmed Dr. Kirk's views, and demonstrated their correctness before the Odontological Society of Pennsylvania. The reader will be justified in the conclusion that the author has written this part in a partisan spirit. As this is manifested so frequently throughout the book, it becomes a question whether the aim has been to establish truth or to uphold certain ideas not generally recognized. While this is well in an argument, it seriously detracts from the value of a publication in which accuracy of statement is most important.

Great credit is due Dr. Bödecker for the careful attention given generally to this work, and it will certainly help, as he says in his preface, to show "that scientific investigations are not neglected in our country," and beyond this it will be of great value as a work of reference to those who continue to devote time to histological work.

In a practical sense, as that is understood here, the book has but little value; in fact, it was not so intended. It contains no treatment of the pathological conditions described, hence its worth as a text-book in colleges cannot be rated very high, but in the library of the student, the thinker, and investigator it must have an important place, and, notwithstanding its many defects, will place the author in high rank among the best workers in his chosen field in this or any other country.

The very full "Literature" of the subjects treated is a valuable addition to the book, and shows much labor devoted to its preparation.

Great credit is due the S. S. White Dental Manufacturing Company for their part of the work. It is fully up to their high standard in every respect.

